# Strengthening capacity to research the social determinants of health in low- and middle-income countries: lessons from the INTREC programme

**DOI:** 10.1186/s12889-017-4399-0

**Published:** 2017-05-25

**Authors:** Nicholas Henschke, Anna Mirny, Joke A Haafkens, Heribert Ramroth, Siwi Padmawati, Martin Bangha, Lisa Berkman, Laksono Trisnantoro, Yulia Blomstedt, Heiko Becher, Osman Sankoh, Peter Byass, John Kinsman

**Affiliations:** 1 Institute of Public Health, University of Heidelberg, Heidelberg, Germany; 2 Harvard Center for Population and Development Studies, Harvard University, Harvard, USA; 3 Centre for Social Science and Global Health, University of Amsterdam, Amsterdam, the Netherlands; 4 Amsterdam Institute of Advanced Labour Studies, University of Amsterdam, Amsterdam, the Netherlands; 5 Universitas Gadjah Mada, Yogyakarta, Indonesia; 6 INDEPTH Network, Accra, Ghana; 7 Department of Public Health and Clinical Medicine, Umeå Centre for Global Health Research, Epidemiology and Global Health, Umeå University, Umeå, Sweden; 8 Institute of Medical Biometry and Epidemiology, University Medical Center Hamburg-Eppendorf, Hamburg, Germany; 9 School of Public Health, Faculty of Health Sciences, University of the Witwatersrand, Johannesburg, South Africa; 10 Faculty of Public Health, Hanoi Medical School, Hanoi, Vietnam

**Keywords:** Social determinants of health, Capacity strengthening, Education, Blended learning, Research methodology

## Abstract

**Background:**

The INDEPTH Training & Research Centres of Excellence (INTREC) collaboration developed a training programme to strengthen social determinants of health (SDH) research in low- and middle-income countries (LMICs). It was piloted among health- and demographic researchers from 9 countries in Africa and Asia. The programme followed a blended learning approach and was split into three consecutive teaching blocks over a 12-month period: 1) an online course of 7 video lectures and assignments on the theory of SDH research; 2) a 2-week qualitative and quantitative methods workshop; and 3) a 1-week data analysis workshop. This report aims to summarise the student evaluations of the pilot and to suggest key lessons for future approaches to strengthen SDH research capacity in LMICs.

**Methods:**

Semi-structured interviews and questionnaires with 24 students from 9 countries in Africa and Asia were used to evaluate each teaching block. Information was collected about the students’ motivation and interest in studying SDH, any challenges they faced during the consecutive teaching blocks, and suggestions they had for future courses on SDH.

**Results:**

Of the 24 students who began the programme, 13 (54%) completed all training activities. The students recognised the need for such a course and its potential to improve their skills as health researchers. The main challenges with the online course were time management, prior knowledge and skills required to participate in the course, and the need to get feedback from teaching staff throughout the learning process. All students found the face-to-face workshops to be of high quality and value for their work, because they offered an opportunity to clarify SDH concepts taught during the online course and to gain practical research skills. After the final teaching block, students felt they had improved their data analysis skills and were better able to develop research proposals, scientific manuscripts, and policy briefs.

**Conclusions:**

The INTREC programme has trained a promising cadre of health researchers who live and work in LMICs, which is an essential component of efforts to identify and reduce national and local level health inequities. Time management and technological issues were the greatest challenges, which can inform future attempts to strengthen research capacity on SDH.

## Background

The World Health Organization (WHO) defines health inequities as “the unfair and avoidable differences in health status seen within and between countries” [1]. Reducing health inequities has been an objective of health policy in many countries and international organisations for decades, with variable results [2, 3]. In 2008, the WHO Commission on Social Determinants of Health (CSDH) [4] provided compelling evidence that the most powerful drivers of health and health inequities are the social conditions in which people are born, live, and work. These are referred to collectively as the social determinants of health (SDH). Several conceptual frameworks that describe pathways by which these determinants can lead to health inequities have been suggested [4–9]. Based on the accumulated evidence for the SDH, the Commission made three major recommendations for action that are needed to reduce health inequities: 1) improve living conditions; 2) tackle the inequitable distribution of money, power and resources that people need to lead a healthy life; and 3) expand the knowledge base on the SDH through monitoring, research, and training [4].

Even though tackling health inequities was one of the objectives of the Millennium Development Goals (MDG) project, at the 2015 deadline it became clear that despite the fact that many countries had made remarkable progress on national-level indicators that were used to measure MDGs, remarkable inequities remained between high-income countries and low- and middle-income countries (LMIC) and between particular groups within countries [4, 10, 11]. In response to this, tackling the SDH inequities has now become a core concern of the post-2015 sustainable development goals. In order to reduce inequities, it is vital that high quality, locally derived, and disaggregated data become available to inform the development, implementation, and evaluation of SDH policies and interventions and that local research cadres are available to provide this data. Research on the socio-economic drivers of health inequity is an emerging field worldwide [12–14], and there was a needed for better training possibilities for SDH research, particularly in LMICs [2, 15]. In response to this need the INTREC (INDEPTH Training & Research Centres of Excellence) collaboration developed and piloted a training programme on SDH research for health- and demographic researchers in LMIC’s in Africa and Asia.

### INTREC

The INTREC consortium consisted of six institutions, five of which are universities (Umeå University in Sweden; Gadjah Mada University in Indonesia; the University of Heidelberg in Germany; the University of Amsterdam in the Netherlands; and Harvard University in the USA) and the sixth being INDEPTH – the International Network for the Demographic Evaluation of Populations and Their Health in Low- and Middle-Income Countries. INDEPTH is an expanding global network of 45 member centres from 20 countries in Africa, Asia, and Oceania, running 52 Health and Demographic Surveillance Systems (HDSS). Each HDSS conducts longitudinal health and demographic research in rural and/or urban populations [16].

The INTREC training program was initially developed for researchers from INDEPTH HDSSs in four African countries (Ghana, Tanzania, South Africa and Kenya) and four Asian countries (Indonesia, India, Vietnam, and Bangladesh) that previously participated research projects on determinants of adult health [17]. Prior to the development of the training program, a situation analysis of existing SDH educational programmes in each target country was performed [15], and learning needs were explored among the potential target groups for the training program [18].

The findings of these assessments were used to develop the INTREC training programme which sought to enable the capacity of researchers to develop and conduct SDH research in their local setting and to share the findings with decision makers. The programme was piloted among a target group of junior researchers working within HDSSs affiliated with the INDEPTH network in 9 countries. In order to select participants for the training, centre leaders of the selected HDSSs were invited to nominate junior research staff to take part. INTREC activities for students from Africa were coordinated by a regional centre in Accra, Ghana and for students from Asia by a regional centre in Yogyakarta, Indonesia.

The programme was developed, organised and taught by consortium partners based on a blended learning approach. Blended learning is a technology-facilitated learning approach in which computer-mediated learning activities are combined with class-room learning while retaining a strong and deliberate role for the teacher in the learning process [19]. It has the potential to draw the maximum benefit from the use of technology (e.g. online courses) while retaining the best features of face-to-face teaching. This makes it ideal for supporting engaging learning activities such as research design and methods, while also allowing students and teachers in different countries to participate and interact.

Detailed information about the curriculum and all teaching materials can be found at www.intrec.info. Briefly, the programme consisted of three consecutive blocks of teaching which ran from November 2013 through to October 2014. Block 1 was an online course of 7 video lectures and assignments, focusing on basic SDH concepts and frameworks. It was taken by 24 students from 9 countries in Africa and Asia over a period of 4 months. The videos and assignments were hosted on the online learning and collaboration platform (“Cambro”) of Umeå University, Sweden. This platform provided information to students about the course and allowed them to communicate with each other and the course coordinator. Students who satisfactorily completed the activities and assignments of Block 1 were selected by INTREC partners to attend the Block 2. Block 2 comprised a 2-week methods workshop, which were taught in a classroom. One week focussed on quantitative approaches to SDH research, the other week on qualitative methodological approaches. Both weeks included sessions on research design, data collection and processing, and practical exercises. For Asian students (*n* = 16) the workshop was held in Indonesia (16 students) and for African students (*N* = 15) in Ghana. Subsequently, a selected number of students who had performed well in the Block 2 workshops were given the opportunity to attend the Block 3 workshop. This final teaching block was a 1-week data analysis workshop held at Harvard University (13 students). Here students were given individual instruction and advice on how to analyse quantitative or qualitative data available in their HDSS, academic writing, preparing a manuscript for publication, and presenting results to an audience of policy makers.

A core component of the INTREC programme involved the evaluation of all aspects of the pilot program in order to provide both specific and general recommendations for future training programmes on SDH. The aim of the current paper is to summarise these evaluation activities to provide an overview of the lessons learned while strengthening research capacity on SDH in LMIC, from the perspectives of individual students, and from a broader, more conceptual level. In doing so, this paper hopes to contribute to addressing the identified training needs for researchers in LMICs so that they are able to produce high quality, locally derived data to inform the development, implementation, and evaluation of SDH policies and interventions.

## Methods

**Fig. 1:**
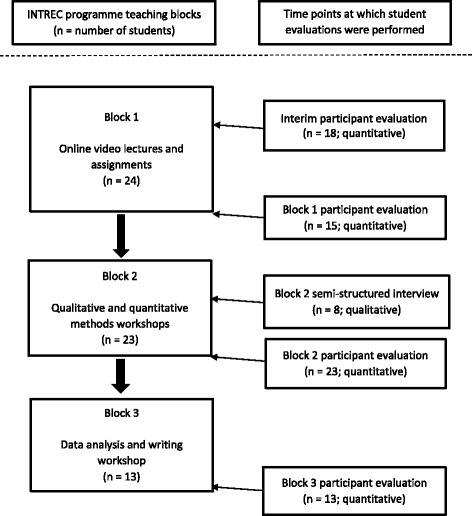
Flowchart of teaching blocks and student evaluations performed

Evaluation of the INTREC programme included formative, ongoing and summative assessment. These activities were conducted over the course of the programme, such that, where feasible, the findings from the evaluation of one teaching block were taken into account in the teaching of the following blocks. To provide a comprehensive evaluation of the student experience within the INTREC training programme, a mixed methods approach was used. This allowed for reflection and comparison of the different teaching and educational methods used within this blended learning programme. Quantitative surveys were administered to all students 1 month after the commencement of Block 1 and following the completion of each teaching block. This was supplemented with face-to-face qualitative interviews performed during the Block 2 workshops, and surveys which included open-ended questions, conducted at the end of all workshops. Figure 1 displays the timing of each student evaluation in relation to the teaching blocks. The evaluation work was concluded by inviting students to reflect on the programme as a whole in an open discussion format during the concluding workshop of the project. Further details of each phase of evaluation are provided below.

### Interim participant evaluation

Four weeks after the start of the online course (Block 1), an interim assessment was performed. Students were invited to complete an online questionnaire where they could express their motivations and goals for participating in the INTREC programme; report on their familiarity and prior experience with online courses; and reflect on the quality of the initial teaching and discussions of Block 1. All students who did not respond to the questionnaire were sent an email reminder 1 week following the evaluation date.

### Block 1 (online course) participant evaluation

Evaluation of Block 1 was conducted through an online questionnaire following the final online lecture and assignment submission, 3 months after the commencement of the training. All students were invited to complete the questionnaire focusing on the structure, content and delivery of the course. This evaluation was supplemented with an assessment of the number of completed assignments submitted by each student (from a total of 6 assignments). The questionnaire consisted of separate rating scales for each of the lectures and assignments, as well as open-ended questions about the time spent doing the course, the student’s experiences of the distance learning approach, and their suggestions for improvements in the future.

### Block 2 (methods workshops) semi-structured interviews of INTREC students

During the mixed methods workshops in Yogyakarta and Accra, all INTREC students were invited to be interviewed in English by one of the INTREC partners who was not involved in the teaching of the workshop (AM). The interviews comprised a semi-structured, mid-programme assessment, aimed at understanding students’ experiences in the programme so far and gathering feedback about the first two teaching blocks. The interviews lasted about half an hour per student, and were recorded with the written consent of the students.

### Block 2 (methods workshops) participant evaluation

At the completion of the Block 2 workshops in Accra and Yogyakarta, an evaluation was conducted through either paper or online questionnaire. All students from both workshops were invited to rate the value of participating in the workshop, suggest what they thought were the most useful aspects, and provide suggestions to improve further training on SDH research. Any differences in the responses between Asian and African students were acknowledged.

### Block 3 (data analysis workshop) participant evaluation

Following the Block 3 data analysis workshop at Harvard, an evaluation form was circulated to all students who attended and thus completed all INTREC training activities. Their feedback was collected through a similar questionnaire to the one used for Block 2, and this was supplemented with an assessment of the final reports that were developed by these students in the following 3 months.

### Data analysis

The responses to closed questions from the questionnaires were analysed using descriptive statistics and reported as percentages. The results from the first two quantitative evaluations were subsequently used to formulate questions for the follow-up semi-structured interviews to give students the opportunity to expand on the difficulties they faced while participating in the INTREC programme.

The responses to the open-ended questions from the questionnaires, and the transcripts of answers elicited by the qualitative semi-structured interviews, were coded and analysed thematically using a form of thematic analysis proposed by Strauss [20]. This involved coding the material, as appropriate, into a set of predefined themes (perspectives on the different training blocks; challenges faced; completion of assignments; and publication), identifying a range of experiences and perspectives from within each theme, and then building a narrative text for each one that encapsulated the main points, with supporting illustrative quotes.

## Results

### Participants

A total of 30 participants were nominated for the INTREC programme, of whom 24 (80%) followed the Block 1 online course. The remaining 6 nominees reported having other commitments and were unable to participate in the programme. The 24 students represented 5 countries in Africa (Ghana, Kenya, South Africa, Ethiopia, Tanzania) and 4 in Asia (India, Indonesia, Vietnam, Papua New Guinea). The students had a range of educational backgrounds and research experience. At the commencement of the programme, all students were working within a HDSS, though their positions ranged from field data collectors to junior research scientists.

All of the initial 24 students were invited to participate in Block 2, of whom 23 (96%) attended and completed the workshop in either Indonesia or Ghana. For Block 3, 13 (54%) students were selected by INTREC partners to attend a week-long data analysis workshop at Harvard University, through which activity these individuals completed all INTREC training activities. Only a limited number of students could be accommodated in Block 3 due to the limited resources available.

### Responses

The initial 24 students were invited to complete the interim evaluation 4 weeks after the commencement of Block 1. Responses were received from 18 (75%) students.

During the Block 2 workshops in Indonesia and Ghana, 23 students who had completed the Block 1 online course participated were invited to be interviewed in person, or via an extended online questionnaire. These interviews were completed by 8 students in Indonesia and 5 students in Ghana.

Of the 24 students participating in Block 1, 15 (63%) provided responses to the Block 1 evaluation questionnaire following the final lecture and assignment. Of the 23 students participating in Block 2, 15 (65%) provided responses to the evaluation questionnaire. All 13 students who attended Block 3 completed the final evaluation.

### Main findings

The primary findings from the different evaluations of the INTREC programme can be summarised in three main themes. These are a) there was strong motivation and interest among the students to perform research on SDH; b) there were a number of technological and practical challenges faced by students, but in spite of these, their evaluation of the teaching methods was very positive; and c) only a small number of assignments, reports, and published manuscripts were produced by the students. These themes are described in more detail below and illustrated with actual quotes from the qualitative interviews.

### Motivation to take part in the INTREC training on SDH

In the interim evaluation, the students identified a variety of reasons for wanting to take part in the INTREC training. More than half (54%) of the students reported having applied SDH concepts in their current (or previous) research before taking the course. Among these students there was widespread recognition that research on the SDH was an “*emerging field that looks beyond biomedical explanations (respondent 2)*” for health and necessary to “*lead to reductions in health disparities (respondent 3)*”. At the time, one-third (33%) of the students reported never having used the SDH framework previously.

During the interviews in Block 2, the students also spoke specifically about their interest in social determinants of health as a new and much-needed perspective for the analyses of their data. They particularly mentioned that the public health training programmes that they had taken did not include an SDH framework.

*“I studied public health in my masters’ programme and consideration of social factors were not included there, I wanted to learn more about it to understand the health issues better.” (A).*

*“SDH is new for me and I want to learn it” (C)*.

*“SDH perspective will help me to understand better issues in our community, like TB or HIV looking at them from social perspective, this is a bit new for us” (R).*

*“In our site in India the caste system is prevalent. So the caste defines where people reside, how they live and their health. Improving their social conditions may really help improve the health conditions. They may be suffering from many diseases due to social conditions, not only economic but also social, education, occupation and the like. We need to learn how to study it.” (A).*

A number of students reported a desire to improve their analytical skills of both qualitative and quantitative data as a major motivator. As most were familiar with data collection and management through their work in their respective HDSS, they hoped that this course could provide an opportunity to develop their scientific skills further. Many students reported that they intended to continue research through a formal PhD programme and that this course would be helpful to improve their ability to write research proposals. Participating in the course was also seen as a way to improve the analysis of data from individual and collective HDSS sites in the future.

*“I’m interested in getting more knowledge in statistics. We have a lot of data at our site but no person to really clean the data, analyse the data and write research proposals. I’m interested in all that.” (B).*

*“I can write a proposal, develop a study and conduct a study, but I need more training with analyses.” (U).*

“…*interested in integrating the social determinants of health and biomedical research so as to understand the areas we need to focus on to improve our public health interventions.” (L).*

*“…acquire and improve my skills in data analysis” (P).*

Overall the students indicated that the leaders of their HDSS centres were supportive of their participation in the programme and interested in their development as SDH researchers.

*“My site will definitely support my research in SDH” (B).*

*“Often my director keeps asking me if I need any support and assistance and where the need be, I do receive support and encouragement” (R).*

*“We have a lot of data but need help with analysing it and writing up the results, we don’t have enough trained personal at the HDSS, that’s why my supervisor recommended me I think” (R).*

*“We have a bit of data at our HDSS site, but problem is how to manage it, managing data is very important and the site needs someone to manage the data and publish the research, we need to help the government to make policy around SDH issues” (A).*

### Student perspectives on Block 1 and challenges faced

Prior to the start of INTREC training, only 35% of students reported having previously taken an online course. However, 75% felt comfortable or very comfortable with taking online courses and no student reported being uncomfortable with this. In general, students reported spending from 3 to 5 h per week on the online course. Most of this time was during their free time or on the weekends (3 to 4 h per week) compared to during working hours (1 to 2 h per week). While 55% of the students found the level of difficulty for the online course “just right”, 45% thought it was “difficult”. The ability and expertise of the students appeared to vary and those without experience in statistics had difficulty with a number of the lectures and assignments. Other students reported having few difficulties even though the topics were new to them.

The majority of students found the online feedback that they received about the assignments from teachers to be quite good (65%) or excellent (20%). They reported that the feedback specifically helped them think critically about their responses, and encouraged them to continue with the course even if they had difficulties. Some students thought that the feedback should be more explanatory and detailed, as well as provided in a timelier manner.

The students were generally positive regarding the distance learning approach. One common suggestion for improvement was to allow the videos to be downloaded for watching at a later time, especially if there was difficulty with the internet connection. Other suggestions for improving the approach included making sample datasets available online for the data analysis assignments (rather than requiring students to use their own), and providing more basic level resources or practical examples for each topic. The main difficulties experienced with the distance learning approach were time management (i.e. students found it difficult not having a focused time to study), and inadequate communication and discussion with the teaching faculty.

From the semi-structured interviews, students reiterated some of the challenges they had while completing Block 1. While all students were confident that their site leaders and other colleagues supported their INTREC training in terms of time, resources and logistical support, it turned out that none of them had been allowed to take time away from their work to do the online course.

*“In the day time I do not have time to work on this project, normally I’d do it in the late evening or on the weekend” (T).*

*“We were not given any special time for this project, I just did it whenever I could” (I) “I’m an early riser, I often did it at 4 or 5 in the morning, before going to an office, I can’t have people at work waiting while I watch the videos”. (A).*

*“We have our families and jobs and it is a bit difficult to find extra time to view the lectures and do the assignments”. (U).*

*“After assignment 4 I received a few complaints that I was spending too much office time on the course work and the assignments.” (G).*

The issue of internet access was also raised by most of the students from Africa:

*“When there is no Internet access they (the site) used to buy credit to recharge my modem-to-connect bundle” (F).*

*“Colleagues at work supported me in downloading some of the recommended articles for further readings” (R). ‬‬.*

In an attempt to mitigate this problem, all HDSS centre leaders and students were also sent USB drives containing the online lectures and resources.

### Student perspectives on Block 2 and Block 3 and challenges faced

Table 1 shows the mean ratings for the workshops in Indonesia (Asia) and Ghana (Africa). Each item was rated in terms of how they were valued by the students on a 5-point scale, where 1 = no value and 5 = very high value. Most items were rated over 4 (high value).

**Table 1:** Mean value ratings of the 2-week Block 2 workshops in Asia (*n* = 8) and Africa (*n* = 15)

	Asia		Africa	
	Quantitative methods	Qualitative methods	Quantitative methods	Qualitative methods
How would you assess the quality of the workshop as a whole?	3.9	4.5	4.5	4.4
How would you assess the way you have been treated in general as a student during the workshop?	4.4	4.6	4.8	4.6
How do you value the course in terms of:				
New and relevant knowledge for your work	4.1	4.2	4.7	4.5
Teaching methods	3.8	3.9	4.3	4.4
The sessions on developing research questions	4.1	4.4	4.5	4.5
The course with regard to exchange of experiences with other students from other countries	4.1	4.4	4.2	4.4

Note: rated on a 5-point scale; 1 = no value and 5 = very high value

Students from both Asia and Africa also reported that the most useful aspects of the Block 2 workshops were: clarifying the concepts of SDH especially after the online course; having time to develop research questions (quantitative and qualitative) on SDH; group work, activities, and discussion; the opportunity for qualitative methods (focus group discussions and observation); and the interaction with researchers from different sites/countries. In addition, all the students seemed to like the friendly and interactive teaching style of the instructors. For example, comparing it to the online course:

*“I definitely like better this workshop, in online course you write a question and then wait for an answer, here it is right there, more interaction, and more useful” (B).*

*“This is a very jovial mood workshop; I’ve never previously attended such a friendly workshop. The instructors are fantastic, they’re doing their job so well that we feel free to ask anything, any question that comes to mind. They do not just stick to the power point presentation as often we see.” (A).*

*“I liked how in the class we look at the examples of different methods, discuss the articles, it’s very friendly and very helpful for understanding” (C).*

“*Interact with the other participants from other sites and provided an opportunity for networking” (G).*

With regard to aspects of the workshop students would like to have changed, many reported that there should be more time in the workshop for hands-on analysis of data using some data sets from HDSS sites. Some students from Africa felt that more of the workshop could have been devoted to more advanced statistics, the interpretation of results, and reporting in scientific publications. Students from Asia thought that more examples of well-conducted SDH research should be used and presented.

“*I have a background in public health, so this information is more like a refresher for me”* (T).

*“I was surprised that after the analyses we did for the lectures 4 and 7 in the online course here we go back to the basics. I really liked the part when we were discussing articles, it helped me and it also helped other students, some are struggling with statistical concepts and these examples are really useful” (I).*

*“This workshop is a bit too mathematical, it would be more useful if we had to practice the data analyses here more”*.

*“I think it would be good to have some kind of ‘dummy data’ and try some analyses here, so we’d have the theoretical and practical aspects together and understand more” (U).*

*“I participated in the quantitative studies before, but I was mainly collecting data, entering into SPSS and doing basic analyses but not understanding much of the discussions of the results done by specialists we collaborated with. I feel like I understand it much better what we did, how they got the p values and correlations, and I think this workshop is a right level for me” (R).*

*“time for workshop was short we needed more days to cover many things and doing more practices” (F).*

*“analysing qualitative data using Open Code 4 by learners could have been a plus” (R).*

With regard to the final teaching block, the evaluations of Bocks 1 and 2 were taken into account and based on these 13 students were selected to attend a week-long data analysis workshop at Harvard University. Each student was assigned a consultant (research scientist) who helped them with their work and all students rated the workshop very highly in terms of teaching quality and support, as well as the relevance for their work. The most useful aspects learnt during the workshop were consistently reported to be around the practical use of data analysis software and how to organize the results in a coherent manner. Students also reported that this kind of workshop could be longer in duration in order to provide more time to complete their work.

*“I had a great consultant… he gave me confidence that I could work well with my dataset. I have a good feel for my data analyses plan now, still have to work on data commands and development of figures but know I can do it”.*

*“The consultant assigned has the interest and support and gave me a lot of insights about the data”.*

*“I liked it very much, I was treated and greeted nicely. I had a very good mentor/consultant. For the first time I had done the statistical analysis on my own”.*

*“A 5-day workshop is too short, I feel I don’t have enough time, however the highly experienced consultants can provide quick, clear and simple feedback, which actually speeds our work”.*

### Completion of assignments and publication of SDH research

**Fig. 2:**
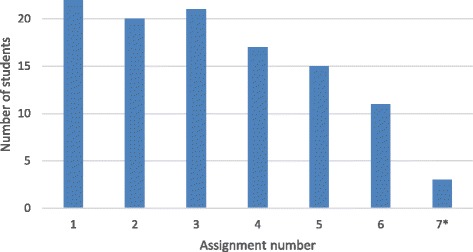
Number of completed assignments from Block 1. *Optional assignment

In order to evaluate the effectiveness of the INTREC programme to produce research on SDH, the number of assignments completed in Block 1 and the number of complete reports or manuscripts were recorded. Figure 2 indicates how many of the assignments were completed by each student during the online course. There were 7 assignments with the final assignment being optional. On average, students completed 4.6 out of the 6 assignments. There were only 11 students (46%) who completed all assignments and 7 (29%) who completed 3 or fewer. The first three assignments were all completed by over 90% of students, but the second three by only 48%-73%.

The main reasons provided by students for not being able to complete all assignments were other work or family commitments, lack of time, or inability to access a suitable internet connection.

In the 6 months following the completion of all INTREC training activities, 6 (46%) of the students had completed a policy brief and two (15%) of these had also submitted manuscripts to peer reviewed journals, which were subsequently published.

## Discussion

There is a global need and demand to strengthen health research capacity in order to collect, analyse, and present policy-relevant data which can improve health outcomes in all regions [21, 22]. Through a combination of online training, face-to-face workshops, and a structured progression from conceptual learning to the practical application of methods, the INTREC programme was specifically designed to strengthen this capacity. The need for a training course on the concepts and methods for researching SDH was widely accepted and supported both by the INTREC students themselves and their HDSS centre leaders [15, 18]. Evaluation of each aspect of the programme has allowed identification of many benefits and challenges of using a blended learning approach to strengthen research capacity in LMIC. The major challenges identified throughout the INTREC programme were time management, the lack of face-to-face instruction in the initial phase, and limited interaction between the students. Further consideration is also needed to identify the best “blend” of approaches to complement the learning objectives, meet the needs of participants, and match the programme’s context to that of local areas within LMIC.

The use of a blended learning approach in this training programme was based on the practicalities involved with training participants from nine different countries across Africa and Asia, as well as its purported benefits over traditional educational approaches. The use of technology as part of a blended learning approach has been widely recognised as an approach that can bridge many of the educational barriers that exist in LMIC [23, 24]. In this instance it was recognised that all participants had access to and familiarity with computer use, meaning there was unlimited scope for technology-mediated communication but limited opportunity for face-to-face instruction. Recent studies have reported that the results of blended learning approaches are similar to, and in some cases better than, traditional classroom approaches [25]. They indicate that blended learning environments are effective for conveying medical knowledge and developing practical competencies [26], as well as promoting a better quality of education in many instances [27]. However, there is little evidence in the literature or from the results of the current evaluation to suggest whether a blended learning approach can be used to increase research output or strengthen overall research capacity.

The evaluation of the Block 1 online course, through questionnaires and semi-structured interviews, suggested that most students required more time to complete the assignments and work through the reading material, especially those students who are not experienced with quantitative analysis and statistical methods. From the results of this evaluation, the main challenge for students participating in such an online course appears to be time management and balancing the demands of their regular work and family life with those of the course. In addition, those students that did not have a strong background in quantitative analysis found some lectures and the assignments very challenging to follow. Further consideration should be given to the prior level of knowledge of students and matching the difficulty of the content to this level. Alternatively, where challenging topics and concepts are introduced, more background information, resources, or exercises could be provided through the online platform as suggested by some students. The development of a blended learning approach to improve research capacity also requires substantial support from the HDSS centres where the students work. While the benefit of the training is apparent for both the individual and the HDSS, further discussion on the demands of the programme are needed to ensure institutional support. Most HDSS centres are also running on limited resources and personnel, making it difficult to allow time for students to follow the programme.

A number of students in Africa also identified that having an inadequate internet connection was a challenge when following the online course. However, this was less of an issue in the Asian countries. Without an adequate internet connection students had limited opportunity for feedback, assignment submission, and interaction. The challenge of bandwidth availability, however, is rapidly being addressed in even the most resource-challenged areas. Bandwidth is expected to increase by 2400% in East Africa in the next decades while costs should be reduced by almost half at the completion of undersea cables currently under construction [28]. Until the technological barriers are overcome other solutions need to be developed which allow all students the ability to participate in such courses.

A lack of face-to-face interaction during the online course was also a challenge highlighted by a number of students. This can contribute to professional isolation and decreased learning experiences [29], and make it difficult to engage participants in discussion [30]. The key element underpinning a blended learning approach is the scope and nature of the communication channels provided to support students, through feedback from faculty members or interaction with other students [26]. The INTREC programme was supplemented with an online educational platform which gave students the opportunity to interact and discuss key issues. Despite not having specific access statistics on the educational platform, there was a general understanding by INTREC faculty and students that this was underutilised. Interaction with other students can be perceived as very beneficial when it occurs in person, such as in the Block 2 and 3 workshops, as it can improve cohesiveness and morale [31]. However, when these interactions are facilitated online their benefit is often not considered as useful [32, 33]. Similar studies have found that the web blog, chat and discussion board were the least utilised technological elements of blended learning programmes, as opposed to emails and web portals, which were more utilised [34]. As there was a substantial reduction in the number of completed assignments in the second half of the INTREC online course, further efforts are needed to provide feedback and encourage interaction among students to reduce this attrition. One possibility would be to ensure that a face-to-face meeting precedes the online part of the programme, in order to increase familiarity among the student group prior to the distance learning part.

In addition to differing results on the most effective components of blended learning programmes, researchers also have differing conclusions on how to determine an appropriate blend between these various learning components [25, 26]. The content and objectives of the course should be taken into account when designing a blended learning programme, but studies are lacking in how content to strengthen health research capacity affects the ideal blend of programme components. While the theoretical and conceptual parts of SDH research may be well suited to distance learning approaches, practical aspects such as data collection, management, and analysis can be considered more suited to face-to-face instruction and discussion. When evaluating the Block 2 and 3 workshops, students from both Asia and Africa found the workshops to be of high quality and value for their work. Gaining practical experience of field work, observation, and analysis was found to be one of the most highly valued parts of the workshop by all students. Students also felt able to improve their practical data analysis skills and develop their research proposals into scientific manuscripts and policy briefs for stakeholders. Most students reported that they would prefer more time during the workshops for analysis of quantitative and qualitative data, using available software. While these approaches seem positive, a caveat must be added that the positive reflections of the INTREC students may be a result of having all expenses covered for their teaching and travel. As such, further consideration is needed to identify approaches to develop practical research skills, many of which are specialised skills such as data analysis or academic writing, in an efficient way which is applicable to LMIC.

Tailoring the INTREC programme to meet country-specific realities, cultures and languages is one area for future programme development [25, 26]. In order to maximise the learning experience, educational materials must be culturally relevant and language appropriate. Tailoring materials to reflect country-specific health care realities and gathering challenging, real-world case studies of effective SDH interventions, should increase interest and relevance in the concepts of SDH research. Material from the INTREC project has been modified with examples of SDH research and interventions in South-East Asia for use in further training at the regional centre in Indonesia. More focus on the evaluation and implementation of different training courses for health research in SDH is needed in order to come to a better understanding of how to maximize impact in this area. In particular, research should move beyond initial knowledge gain and look towards long-term application of knowledge and skills. There is also a need for more experimental design to illuminate the impact and effectiveness of individual components within the blended learning approach.

## Conclusions

The INTREC programme has provided numerous insights into how health research capacity on SDH may be strengthened in low-resource settings. Improving time management of the learners, making a wider range of teaching resources available, and finding appropriate technological solutions appear to be key to improving such teaching programmes. The programme has trained a promising cadre of individuals who live and work in LMICs, which is an essential component of efforts to identify and reduce national and local level health inequities. While the student experience was mostly positive, the challenges faced in this programme can help to inform future attempts to strengthen research capacity on SDH.

## Abbreviations

MDG: Millennium Development Goal
INDEPTH: International Network for the Demographic Evaluation of Populations and Their Health in Low- and Middle-Income Countries
INTREC: INDEPTH Training & Research Centres of Excellence
SDH: Social determinants of health
LMIC: Low- and middle-income countries
WHO: World Health Organization
CSDH: Commission on social determinants of health
HDSS: Health and Demographic Surveillance Systems
